# A millennium-long reconstruction of damaging hydrological events across Italy

**DOI:** 10.1038/s41598-019-46207-7

**Published:** 2019-07-10

**Authors:** Nazzareno Diodato, Fredrik Charpentier Ljungqvist, Gianni Bellocchi

**Affiliations:** 1 Met European Research Observatory – International Affiliates’ Program of the University Corporation for Atmospheric Research, Via Monte Pino snc, 82100 Benevento, Italy; 2 Department of History, Stockholm University, 106 91 Stockholm, Sweden; 3 Bolin Centre for Climate Research, Stockholm University, 106 91 Stockholm, Sweden; 4 Department of Geography, University of Cambridge, CB2 3EN Cambridge, United Kingdom; 5 UCA, INRA, VetAgro Sup, Unité Mixte de Recherche sur l’Écosystème Prairial (UREP), 63000 Clermont-Ferrand, France

**Keywords:** Palaeoclimate, Hydrology

## Abstract

Damaging hydrological events are extreme phenomena with potentially severe impacts on human societies. Here, we present the hitherto longest reconstruction of damaging hydrological events for Italy, and for the whole Mediterranean region, revealing 674 such events over the period 800–2017. For any given year, we established a severity index based on information in historical documentary records, facilitating the transformation of the data into a continuous time-series. Episodes of hydrological extremes disrupted ecosystems during the more severe events by changing landforms. The frequency and severity of damaging hydrological events across Italy were likely influenced by the mode of the Atlantic Multidecadal Variability (AMV), with relatively few events during the warm Medieval Climate Anomaly dominated by a positive phase of the AMV. More frequent and heavier storms prevailed during the cold Little Ice Age, dominated by a more negative phase of the AMV. Since the mid-19^th^ century, a decreasing occurrence of exceptional hydrological events is evident, especially during the most recent decades, but this decrease is not yet unprecedented in the context of the past twelve centuries.

## Introduction

Both climate model simulations and physical theory suggest that precipitation patterns will change with global warming and, broadly, confirm to the “wet-gets-wetter and dry-gets-drier” paradigm^1,2^. In reality, temperature–hydroclimate relationships increasingly appear to be more complicated and spatially more heterogeneous than this simplistic pattern^3–6^. At the same time, palaeoclimate proxy reconstructions have revealed that recent hydroclimate changes are not yet unprecedented in the context of the past one to two millennia^7–10^. Still, many regions have experienced an increase during recent decades in daily and/or hourly precipitation extremes and thus an increased flood risk^11–13^. Natural palaeoclimate proxy archives are lacking the temporal resolution necessary to resolve such events. Thus, it is unknown if the recent increase in daily and/or hourly precipitation extremes is unprecedented or not in a long-term context. Moreover, the lack of such data has made it challenging to assess the long-term relationships between temperature changes and changes in daily or hourly precipitation extremes.

Historical documentary data, from regions where long such archives are available, offer a unique possibility to reconstruct past climate events down to a daily or even hourly basis^14^. Reconstructions from such documentary archives of past precipitation extremes are important for the understanding of the variability and changes of floods and landslides, but also to gain better insights into their impacts on ecosystem functioning and societal impacts^15^. Natural climate variability induces considerable inter-decadal fluctuations in stormy events^16^, including so-called damaging hydrological events (DHE)^17^, not least in the coastal and mountainous areas of the Mediterranean region. Studies of past climate in Europe^9,18–24^ have documented a long history of warmer and colder climate as well as wetter and drier periods, alternating repeatedly and often abruptly, with a profound influence on shaping multiple damaging hydrological events^25–29^. For instance, Bradley *et al*.^30^ and Ljungqvist *et al*.^10^ identified past periods of Mediterranean hydroclimate showing similarities to that of the present. However, the millennium-long evolution of hydrological extreme events for the Mediterranean Basin has hitherto only been studied to a very limited extent^31–35^.

To fill in this gap, we assessed historical documentary data describing DHEs in Italy during the period 800–2017. Historical documentary data, including diaries, can provide high-resolution meteorological as well as social, cultural and economic information. They can often also provide information about social vulnerability to climate extremes and allow for a direct comparison with contemporary climatology^36^. In addition to Italian archival and library research, a number of literary sources were accessed through a web-engine search of digitized old books (https://books.google.com), generating ~100,000 bibliographic entries. In this first comprehensive bibliographic search, only ~1,000 entries met the criteria of the keywords *rainfall*, *storm*, *flood* and *alluvial*, in addition to some Latin locutions, and were chosen for careful reading; in turn, only 150 literary works were found to be useful for the region and period of interest here. The data of interest from the documentary sources were retrieved by transforming the information from the documentary sources into numerical values on an index scale (see Methodological criteria and Catalogue of Storm Severity Index (SSI) in Methods and Supplementary Information, respectively). We thus created annual indices – a Storm Severity Index Sum (SSIS) – from the sum for SSI of seven Italian regions (following either administrative or natural borders). This process presents a significant challenge, requiring a dynamical understanding of the historical sources in question in addition to a deep knowledge of the regional climates and a familiarity with the relative strengths and weaknesses of each source type. For this reason, a procedure called *weather hindcasting*^37^ was applied to become familiar with well-documented anomalies within the instrumental period prior to analysing analogous cases in the pre-instrumental past. In this way, a scoring system was established to grade SSI per region (calculated in the period of the year between October and April), equal to 0 (normal), 1 (stormy), 2 (stormy with a few floods), 3 (stormy with big floods) and 4 (extraordinarily stormy with very large floods)^38^. Storms occurring during the warm season (May to September) were not considered here as these storms generally only affect restricted areas of the present-day Italian territory and, as such, are not representative for the reconstruction of storminess in this study.

## Environmental Setting

**Figure 1:**
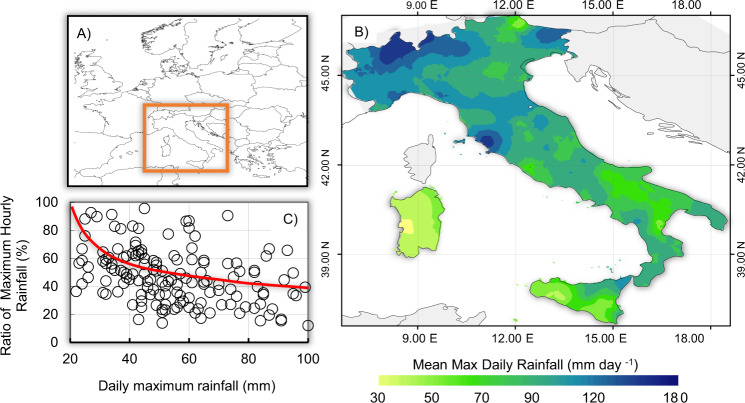
Environmental setting and nature of precipitation. (**A**) The study region. (**B**) Spatial pattern of the mean maximum daily rainfall with inverse distance weighting interpolation over the period 1994–2003, and (**C**) ratio of maximum recorded hourly rainfall versus maximum daily precipitation for Italy, with natural-log interpolation function (red curve) based on a dataset from the *Rete Agrometeorologica Nazionale*^95^.

Cyclonic circulations – due to their frequency, duration and intensity – play an important role in the weather and climate system over the entire Mediterranean Basin; a large spectrum of environmental variables and phenomena are associated with cyclones in this region. Wind regime, air pressure, temperature, cloudiness, precipitation, thunderstorms, floods, waves, storm surges, landslides, avalanches, air quality and even fog and visibility in the Mediterranean are influenced by the formation and passage of cyclonic disturbances^39^. The central Mediterranean region, although located to the south of the main Atlantic storm track that more directly affects northwestern Europe (Fig. 1A), is quite frequently subjected to sudden events of extreme and adverse weather, often with large societal and economic impacts. The northernmost part of the central Mediterranean region is particularly stormy^40^, though DHEs are common in central and southern Italy^26^. This manifests the fact that the maximum daily rainfall of the less stormy, more southern, hydroclimatic areas is still capable of causing damages (Fig. 1B). For instance, in areas with rainfall less than 50 mm d^−1^, the ratio is typically >50%, which means that more than half of the heavy daily rain falls in very short intense-storms (red curve in Fig. 1C).

### Completeness of the data set

We revealed the presence of 674 damaging hydrological events in the assessed historical documentary data. Sorting these events into classes of severity gives 232 *stormy events*, 292 *very stormy events*, 112 *great stormy events*, and 38 episodes of *extraordinary stormy events*, distributed across seven regions of Italy (Table 1). They include administrative regions (Marche, and Calabria and Sicily together), river basins (Adige in northern Italy, Arno and Tiber in central Italy, and Calore in southern Italy) and sets of sites (“hot-spots”) whose exact location remain unknown, or are known and are different each time but do not cover the other regions, for which storm events are noted in historical sources. In this way, we accounted for areas with of different size  in order to represent hydrological and meteorological phenomena operating at different spatial scales (e.g., the consequences of a heavy thunderstorm are not the same if the event occurs in a small basin or in a large region). Other hydrographic basins/regions of Italy have not been included in this study as documentary data from them are not providing sufficiently detailed information extending back into early medieval times. For instance, ancient sources from several regions of southern Italy are only fragmentarily available. Moreover, two geographically adjacent regions, Calabria and Sicily, were merged with the aim to ensure the continuity of the sources between older and more recent times.

**Table 1:** Number of storm severity index (SSI) classes for each geographical region.

Severity classes (SSI)	Marche Region	Arno Basin	“Hot-Spot” (Italy)	Tiber Basin	Calore Basin	Adige Basin	Calabria + Sicily
1	21	31	50	30	30	30	20
2	51	47	65	40	20	43	26
3	12	16	20	24	7	15	18
4	3	5	6	13	4	4	3

**Figure 2:**
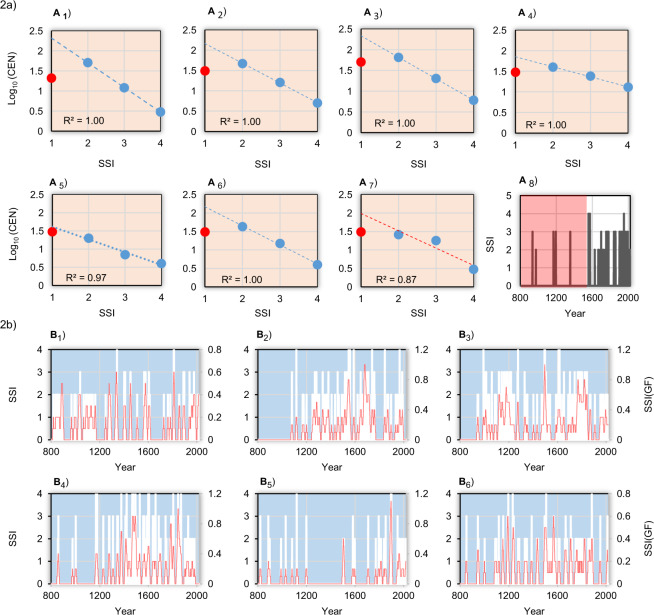
Cumulative distribution of the logarithm of number of storm events versus their storm severity index (SSI) and relative time-series during the period 800–2017 in Italy. (Panel a) A_1_ Marche region, A_1_ Arno river basin, A_2_ Hot-Spots, A_3_ Tiber basin, A_4_ Calore basin, A_5_ Adige basin, and A_6_ Calabrian-Sicily regions. In A_7_ note that for this last area, the catalogue result not complete (data points are not aligned along the linear regression), and this was due to shortage of information during the period 1200–1500 (pink area of the SSI-time series. Red points in the scatterplots represent the storm event with SSI = 1. (Panel b) As in a, but for time-series of SSI, respectively.

However, there can be different factors of uncertainty in the classification of the storm data. It is well-established that there is a tendency in historical documentary data to underestimate small events with isolated storms, especially when they occurred in an area with poorer communication infrastructures^41,42^. To overcome some uncertainties, we established a “reasonable criterion” with respect to the recorded SSI events. This was done by verifying, for each region, the scale-invariance in the relationship between the number of events larger than the storm strength events and the same strength events^43^. The completeness analysis was formalized with the relationship between the cumulative number of events (*CEN*) and SSI values within the range 1 ≤ SSI ≤ 4, as follows^43^ (Eq. 1):

$$\,lo{g}_{10}(CE{N}_{i,j})=a+b\cdot SS{I}_{i,j}\,{\rm{with}}\,\,i=1,\ldots ,\,\,4\,{\rm{and}}\,\,j=1,\ldots ,\,\,7,$$

where the SSI is the storm severity index by severity class (*i*) and region (*j*). Negative slopes in Fig. 2 reflect the principle of a progression towards less frequency as storm events become larger.

A Pearson correlation coefficient *r* = 1.00 is expected, with probability to reject *r* = 0 equal to *p* < 0.01. The catalogue concerning the Calabrian-Sicilian area, for which a shortage of information is evident during the period 1200–1500 (pink area in Fig. 2a,A_1_ and A_8_), represents an exception. For this reason, the Calabrian-Sicilian series was not included in the final catalogue, so that the storm events during the period 800–2017 may be assumed significantly scale-invariant and complete only for the 395 events that, within the range 2 ≤ SSI ≤ 4, are described in qualitative terms as very, great and extraordinary storms. The remaining 212 events with SSI = 1 (red points in the scatterplots) are not fitted by the regression line drawn in Fig. 2a,A_1_–A_7_. Their number is much smaller than required by Eq. (1), probably because many of these lowest energetic events escaped detection in the past. The events with SSI = 1, classified as stormy events, have been discarded from temporal analysis because they are not representative of the entire catalogue within the period 800–2017. Figure 2b,B_1_–B_6_ illustrates the time-series of single regions that have passed the completeness test, with the millennial-long evolution of SSI values (white histograms) and their respective 31-year Gaussian filtered series (red curve).

From a practical point of view, the invariance of the temporal scale is verified when – having taken a sufficient time *t* within the series – this must be subject to the “law” according to which the very extreme phenomenon (e.g., catastrophic flood) is expected to happen fewer times than the slightly less extreme event (e.g. major flood), and so forth, down to light events (e.g., just a storm) that are expected to occur frequently. It may happen that even apparently continuous series do not fully satisfy the criterion of temporal homogeneity, and then the invariance of the time-scale. As an example, a continuous and apparently homogeneous series may contain only storm events belonging to the low and medium classes in ancient times compared to the most recent part of the series, where events of different magnitude are present because of the availability of richer documentary sources. In such cases, it must be concluded that the series, although presenting a temporal continuity of events, is not uniformly representing the relative classes of the same events, which are not homogeneously distributed across the series. As a consequence, the scale invariance is not respected and the series has to be discarded for further analysis.

### Regional reconstruction of damaging hydrological events

With help of the historical sequence of stormy winters, it is possible to study the influences of climate variability on rainstorms over the period 800–2017. We focus particularly on the relatively warm Medieval Climate Anomaly (MCA, here 800–1249), the cold Little Ice Age (LIA, here 1250–1849), and the period of recent warming (RW) (since 1850). The recurrence of expected damaging hydrological events may be fundamentally explained by repeated occurrences of a storm severity index sum. In order to identify possible trends and oscillation in hydrological events, the time-series was filtered by a low-pass 11-year Gaussian function – SSIS (GF) – designed for this purpose following ref.^44^.

**Figure 3:**
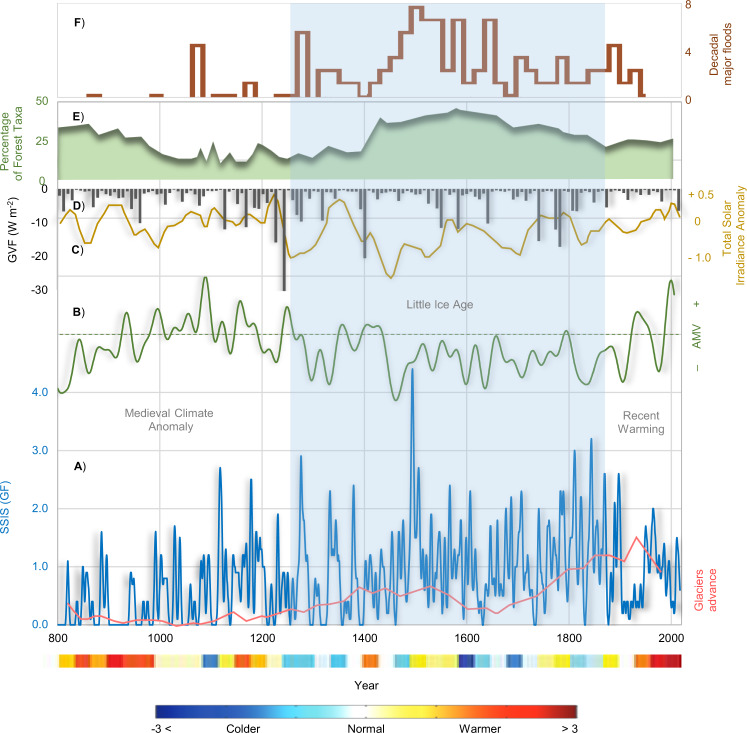
Overview of several environmental and climate patterns during the past twelve centuries (800–2017). (**A**) Evolution of the smoothed 11-year Gaussian filter of storm severity index sum – SSIS(GF) – in Italy (blue curve) with retreat/advance of the Langjökull glacier (Iceland) reconstructed from the Hvítárvatn lake varves^96^ (red curve). (**B**) Atlantic Multidecadal Variability (AMV, green curve)^45^. (**C**) Total solar irradiance anomaly from the value of the Physikalisch-Meteorologisches Observatorium Davos (PMOD) composite during the solar cycle minimum of the year 1986 (1365.57 W m^–2^)^51^. (**D**) Global volcanic forcing^53^. (**E**) Percentage arboreal pollen record (green shape) averaged between central and southern Italy^46,97^, respectively, and (**F**) Hydrological variability for the Po River Basin (brown curve) reconstructed by foraminiferal δ^18^O record-sediment core^33^. The coloured bands of surface air temperature anomalies with regard to the 1961–1990 climate baseline for Europe^61^, at the bottom.

In most of our regional reconstructions, there were only a few stormy years and major floods occurring during the first three centuries (800–1099). During this period, a positive phase of the Atlantic Multidecadal Variability (AMV)^45^ was mostly dominant. Regional precipitation was well below the long-term average and temperatures remained above the long-term average^46^. Furthermore, Fig. 3A indicates varying levels of inter-annual variability in flood events between *c*. 1200 and 1400, when the pollen counts were extremely low; the pollen data clearly suggest that large areas were highly deforested as early as the 10^th^ century^46^ (Fig. 3E). The deforestation may have favoured the occurence of multi-annual flooding pulses of moderate intensity until reforestation occurred during the 15^th^ century. This was accompanied by damaging hydrological events in Italy^47^ and much of Europe^48,49^.

Sutton and Dong^50^ argued for the existence of a causal link between a positive phase of the AMV and drier conditions over the Mediterranean Basin, reflected in less precipitation extremes during the MCA and the RW periods (Fig. 3A,B). In contrast, in the 13^th^ century the AMV shows a decisive shift towards a negative mode, coinciding with the onset of the LIA. Possible additional climate mechanisms governing the frequency of torrential floods in the central Mediterranean region during the successive colder phase of the LIA are long periods of reduced solar activity^51,52^ and enhanced volcanic activity^53^ causing large-scale cooling (Fig. 3C,D). These concurrent climatic factors likely amplified cooling, making it very difficult to separate volcanic and solar forcing^54^. However, Xoplaki *et al*.^55^ found that the variability in multidecadal mean precipitation and drought in the central and eastern Mediterranean cannot be explained by external forcing agents (e.g. solar and volcanic forcing). Rather, they are mainly driven by internal climate dynamics. Hurrel^56^ and Sanchez-Gomez *et al*.^57^ found that negative phases of the North Atlantic Oscillation (NAO) are associated with increased precipitation in southern Europe. However, Barrera-Escoda and Llasat^58^ found that the relationship between the NAO and floods is not as strong, due to the important role of mesoscale factors in heavy precipitation for the Mediterranean region. Diodato and Bellocchi^59^ found no support for a relationship between extreme rainfall at Naples (40°50′N, 14°15′E) and NAO dynamics, suggesting oscillations induced by combined atmosphere and ocean influences as reflected in the AMO and Pacific Decadal Oscillation (PDO) indices. During ~1400–1600 floods reached their maximum, before decreasing temporarily and increasing again from ~1700 onwards, in correspondence with a gradual decrease of woodland relative to cropland (Fig. 3A,E).

Hodgkins *et al*.^60^ found that the occurrence of major flooding in Europe and North America often varied driven by the AMV. During the recent warming, the AMV has reverted to a positive mode, with decreasing storms and a sudden decline of floods in Italy during the 20^th^ century. Extraordinary and great stormy winters are very isolated events during the 20^th^ century (e.g., Arno River flooded in 1966), with mostly SSIS(GF) <2 throughout the 20^th^ century and the beginning of the 21^st^ century. We compared our twelve century-long reconstruction of damaging hydrological events with independent datasets, including the occurrence of major floods at decadal scale for Po River Basin^33^ (Fig. 3F, brown histogram). In Fig. 3E (green shape), we also show the percentage of arboreal pollen averaged between central and southern Italian sites^46,61^, respectively. These fluctuations are in general agreement with our DHEs reconstruction throughout the past twelve centuries. This adds additional strength to our work because the time-series from which the data originate come from are totally independent from the documentary data used here. Another factor highlighting that the reconstructed DHE fluctuations indeed are genuine is a reconstruction of extreme hydrological events in the Po River Basin. In addition, at longer time-scales stronger the damaging hydrological events appear to correspond reasonably well with glacial advance phases in Iceland occurring during the LIA (Fig. 3A, orange curve).

### Variability, frequency distribution and persistence patterns

**Figure 4:**
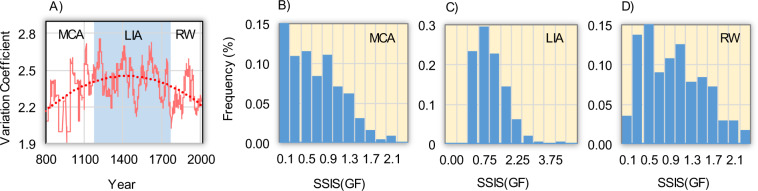
Multidecadal variability of variation coefficients of the SSI for all the regions with (**A**) moving-window of 31-years with an over-imposed polynomial dotted curve, (**B**) relative percentage frequency of SSIS(GF) distribution pattern over 800–1250 (MCA: Medieval Climate Anomaly), (**C**) 1250–1849 (LIA: Little Ice Age), and (**D**) 1850–2017 (RW: Recent Warming).

The variability of storminess measures to what extent the annual storms deviate from their average value, and plays an important role in the occurrence of damaging extreme hydrological phenomena. Here, we present the coefficient of variation calculated for all the six regions with a 31-year moving window (Fig. 4A, orange curve) with the respective long-term trend interpolation (red dotted curve). It is possible to identify, in this long-term trend, a greater inter-annual variability present during the LIA (1250–1849), as compared with the MCA (800–1249) and the RW (1850–2017), as well as the presence of some important multi-decadal oscillations.

It is difficult to detect signals of climate change in the modulation of convection associated with the extreme storm events across Italy because there is often large spatial variability in their occurrence^62^. The histograms of the relative frequency of storm events indicate that the MCA and the RW (Fig. 4B,D) present a generally similar pattern as opposed to the LIA (Fig. 4C). During the MCA and RW periods some variability in the form of quasi-multimodality displaces the distribution from the symmetrical pattern (Gaussian shape) observed during the LIA. The similarity and multimodality of the distributions in MCA and RW periods are indicative of situations where precipitation largely is dominated by storm-like systems, bringing DHEs across Italy. We hypothesize that – contrary to during the warmer medieval and recent times – relatively homogeneous (in space and time) precipitating systems crossed the Mediterranean area during the cold LIA^63^. Under these conditions, the domination of local and non-homogeneous effects, typical of the warming phases^62^, could aggravate critical climatic-environmental situations in the most vulnerable parts of the Mediterranean Basin, where a certain inability to measure intense and unpredictable rainstorms persists, owing to insufficiently distributed rain gauge networks^64^ mostly concentrated over land masses^65^.

## Conclusions

We have presented the first, annually resolved, millennium-long (800–2017) reconstruction of the variability of hydrological extreme events across Italy. The LIA appears to have been a period of increased regional storminess, with a much more homogeneous distribution of storm events compared to the storm patterns occurring during medieval and recent times. Without providing conclusive evidence regarding the causes of the observed changes in the reconstructed frequency and severity of storm events, we suggest that the different patterns of storminess during the past twelve centuries can be linked to large-scale changes in the modes of climate, in particular the AMV. Focusing on climatology and inter-annual to inter-decadal variability, this study provides a more detailed analysis and a useful insight into the Mediterranean hydrology for which past records reflect wet and dry episodes alternating over decadal to centennial time-scales with major, abrupt transitions, reflecting changes in atmospheric circulation^66^. In this way, historical records alongside modern observation data offer a valuable chance to extend information about storms and floods, and how their impacts propagate over time and space. This study offers a spectrum of evidence to orient further research towards sub-regional and local analyses and thereby increasing the knowledge of the spatial variability of the features of different DHEs. Ultimately, this would help to facilitate a better knowledge of the complex nature of the flood challenges and their management^67^.

## Methods

### Documentary sources

In much of the Mediterranean region, as elsewhere in Europe, the proportion of surviving documentary sources increases during the medieval period, enabling the development of climatic indices^37,59^. Instrumental measurements of natural phenomena are lacking prior to the late 17^th^ century and we rarely have regular meteorological indications^68^. Historical information, including such obtained from diaries, are thus the source avalible of quantitative meteorological, social, cultural, and economic information, providing knowledge about social vulnerability to climate extremes and allowing for direct comparison with contemporary climatology^36^. However, research of this type is difficult, and require an interdisciplinary approach^41^, based on collaboration between historians, geographers and climatologists^69^.

The study of the most famous fluvial floods suffered by the urban population of the Middle Ages offers the possibility to conceptually overcome a too rigid distinction between natural disasters and disasters produced by human agency. In fact, contemporary people perceived the occurrence of flood events as both the result of natural factors and human actions, without distinguishing between them. Disasters of this kind to the detriment of the cities strongly impressed the people of the time. Reporters, poets, magistrates of government, as well as religious authorities, left a substantial documentation made of both narratives and legislative provisions^70^. In many ancient sources or sources referring to original documents, information is available in Latin, and there are several declinations for the term *storm* and its damaging hydrological events (DHEs), such as *diluvium, inundatio, excrescentia, fluminum* and related composite locutions such as *magnae pluviae, aqua maxima, tanta aquorum, inundation abundavit, impetus & aquarum multitudine*.

All text passages relating to climate were made machine-readable, for a rapid comparison of data in space and over time^71^. A first key reference reporting ancient chronicles is the book of Sethus Calvisius (1556–1615), a German chronologist and astronomer, written in Latin, *Opus Chronologicum*^72^, reporting a detailed and extended list of the natural hazards and extreme climatic events since Antiquity. Later, Father Secondo Lancellotti (1583–1643) published an important book in Italian in Venice^73^, listing the events with the year as heading on the side, and the source is mentioned but much shortened. Other records referring to medieval chronicles capturing climatic information are mainly found in the Annals of Di Meo^74^, which report a wide variety of DHEs for southern Italy. Among other literary sources of the Italian Middle Ages, there are various medieval chronolicals, such as the *Cavense*, published in 1754 by Francesco Maria Pratilli^75^ (1689–1763), and the *Chronica of Salimbene Parmensis*^76^. An important and extended documentary source is the *Monumenta Germaniae Historica*, published by Georg Heinrich Pertz (1795–1876)^77^ and available in different editions (1826, 1844, 1859 and 1866). It includes a full set of carefully prepared sources covering a period of time ranging from the fall of the Western Roman Empire (476) to the 16^th^ century. Other specific information on abnormal rainfall and floods in Italy can be found in the *Annali of Geografia Fisica*^78^.

Most of the research in historical climatology in Europe focus on the Early Modern Period (*c*. 1500–1800), partly due to lack of documentary data from earlier periods for most of the continent. They have established many of the methods and procedures that have become a standard in this discipline^37^. For instance, the keeping of weather diaries in Europe became a scientific practice from the late-15^th^ century, i.e. from the beginning of the Early Modern Period^79^. Among these, there are the diaries of Moio and Susanna, who report, for the South of Italy, information on both climate and famines from 1500 to 1769^80^. Then, there is also a wide range of narrative texts in which the information cannot be found except in generic historical sources, that is, broader discourses touching the secular affairs, as well as political, medical and religious ones^81^. They seem to recommend merging the natural and social sciences^82^. These texts are generally available from the Middle Ages onward, for both southern and northern Italy. It should be taken into account that the major centres of literacy, besides the Curia in Rome, first were located in Monte Cassino (41°29′N, 13°48′E), then in Benevento (41°08′N, 14°47′E), Bari (41°08′N, 16°52′E) and San Vincenzo al Volturno (41°38′N, 14°05′E)^83–85^. In central and northern Italy, Veneto documents^41,86–89^, and others sources from the Arno^70^ and Tiber River basin^90,91^, also provide a unique opportunity for reconstructing a long time-series of extreme events that have characterized the climate over the past twelve centuries. The southern-most Italian areas are, however, not well-represented in the medieval chronicles and we do not know whether these regions were lacking reported events or actually were not subject to DHEs during this period. Relevant sources for other Italian basins/regions are available from several basins/regions, but they were not aggregated into our study as they do not extend back into the early Middle Ages. For instance, the study by Roccati *et al*.^92^ contains a useful hydrological analysis for a Ligurian River Basin (Entella River), containing detailed hydrological information extending back to 1758. In the same region, documentary sources for the Bisagno River date back to 1400 but not further.

### Criteria for collecting and interpreting the information

We have taken into account all information with a direct bearing on weather effects, such as stormy seasons that are related to extraordinary or continuous rainfall, and the effects on the terrestrial system, such as storm that have caused periods of floods on agricultural lands, and damages to transport infrastructural networks. These data were referred for seven areas representative of Italian territory: (1) the Adige river basin, (2) the Arno basin, (3) the Marche region, (4) the Tiber basin, (5) the Calore basin, (6) the Calabrian and Sicily regions, and (7) the entire territory of Italy, for events that have not a precise indication of local injuries. For each of these areas, a Storm Severity Index (SSI) was developed. Creating the SSI presents a significant challenge, requiring a dynamical understanding of the evidence base in addition to a deep knowledge of the regional climates and familiarity with the strengths and weaknesses of each historical source.

We set a grade SSI per year and for each region (see Supplementary Information), equal to 0 (normal), 1 (stormy event), 2 (very stormy event), 3 (great stormy event) and 4 (extraordinary stormy event) as following the explication^38^:

**Normal** means average or storm passed unobserved, without comments about the severity of its impact on the society and economy.

**Stormy**, an event is considered stormy if the rainfall was intense, and only limited damage is recorded. No floods are recorded.

**Very stormy** was classified when intense rainfall occurred with some floods.

**Great stormy** is an extreme event, with severe and large floods and with agricultural works are suspended, and urban communication interrupted.

**Extraordinary stormy** is characterized by sporadic very extreme events, with a low (centential-scale) recurrence rate. These extreme events affect, at the same time, several river basins, killing people, animals and felling trees.

We based the reconstruction of annually-resolved time-series on the year of each event without assessing for daily/monthly regimes of events. The creation of an annual index was accomplished by the sum of SSI of each region, make up the Storm Severity Index Sum (SSIS)^93^. This procedure was repeated several times in order to reduce inhomogeneities. The study was based on the systematic and critical analysis of data on the above-mentioned phenomena offered by Italian documentary sources for a period covering the period 800–2017. For this area and for this period we have many historical sources, especially in Italy, chronologically compiled from the 9^th^ century. For most of the information, it was possible to make an event check by considering more than one documentary source. It was also possible to contextualise the storm events with other types of historical events (e.g., of economic, social, agricultural, religious nature). In this way, the reliability of information can be assessed by sheding light on the issue of climate relations and extreme events, looking beyond the quantitative data, and seeking alternative sources in diaries, chronicles and local stories that generally provide considerable attention to climate, thus conditioning the social, economic and agricultural life^94^. In this way, the analysis can be greatly enriched and not limited to climatic events alone.

## Supplementary information

Supplement containing the data from the reconstruction
