# Author Correction: A millennium-long reconstruction of damaging hydrological events across Italy

**DOI:** 10.1038/s41598-020-66765-5

**Published:** 2020-06-16

**Authors:** Nazzareno Diodato, Fredrik Charpentier Ljungqvist, Gianni Bellocchi

**Affiliations:** 1 Met european Research Observatory – international Affiliates’ Program of the University corporation for Atmospheric Research, Via Monte Pino snc, 82100 Benevento, Italy; 2 Department of History, Stockholm University, 106 91 Stockholm, Sweden; 3 Bolin Centre for Climate Research, Stockholm University, 106 91 Stockholm, Sweden; 4 Department of Geography, University of Cambridge, CB2 3EN Cambridge, United Kingdom; 5 UcA, inRA, VetAgro Sup, Unité Mixte de Recherche sur l’Écosystème Prairial (UREP), 63000 Clermont, Ferrand France

Correction to: *Scientific Reports* 10.1038/s41598-019-46207-7, published online 10 July 2019

In the Supplementary Information file originally published with this Article, the eighth column titled ‘G’ of the table was omitted and the final column titled ‘SSIS’ was incorrectly formatted. These errors have been corrected in the Supplementary Information that now accompanies the Article.

